# The Paris System for Reporting Urinary Cytology: A Meta-Analysis

**DOI:** 10.3390/jpm12020170

**Published:** 2022-01-27

**Authors:** Ilias P. Nikas, Svenja Seide, Tanja Proctor, Zoi Kleinaki, Maria Kleinaki, Jordan P. Reynolds

**Affiliations:** 1 School of Medicine, European University Cyprus, Nicosia 2404, Cyprus; zoedol@hotmail.com (Z.K.); mariakleinaki@gmail.com (M.K.); 2 Institute of Medical Biometry, University of Heidelberg, 69120 Heidelberg, Germany; seide@imbi.uni-heidelberg.de (S.S.); proctor@imbi.uni-heidelberg.de (T.P.); 3 Internal Medicine Department, General Hospital of Nikea, 18454 Athens, Greece; 4 Department of Laboratory Medicine and Pathology, Mayo Clinic, Jacksonville, FL 32256, USA; reynolds.jordan@mayo.edu

**Keywords:** bladder cancer, urothelial carcinoma, urothelial neoplasia, cytopathology, urine, diagnostic accuracy, sensitivity and specificity, risk of high-grade malignancy (ROHM), tumor, pathology

## Abstract

The Paris System (TPS) for Reporting Urinary Cytology is a standardized, evidence-based reporting system, comprising seven diagnostic categories: nondiagnostic, negative for high-grade urothelial carcinoma (NHGUC), atypical urothelial cells (AUC), suspicious for high-grade urothelial carcinoma (SHGUC), HGUC, low-grade urothelial neoplasm (LGUN), and other malignancies. This study aimed to calculate the pooled risk of high-grade malignancy (ROHM) of each category and demonstrate the diagnostic accuracy of urine cytology reported with TPS. Four databases (PubMed, Embase, Scopus, Web of Science) were searched. Specific inclusion and exclusion criteria were applied, while data were extracted and analyzed both qualitatively and quantitatively. The pooled ROHM was 17.70% for the nondiagnostic category (95% CI, 0.0650; 0.3997), 13.04% for the NHGUC (95% CI, 0.0932; 0.1796), 38.65% for the AUC (95% CI, 0.3042; 0.4759), 12.45% for the LGUN (95% CI, 0.0431; 0.3101), 76.89 for the SHGUC (95% CI, 0.7063; 0.8216), and 91.79% for the HGUC and other malignancies (95% CI, 0.8722; 0.9482). A summary ROC curve was created and the Area Under the Curve (AUC) was 0.849, while the pooled sensitivity was 0.669 (95% CI, 0.589; 0.741) and false-positive rate was 0.101 (95% CI, 0.063; 0.158). In addition, the pooled DOR of the included studies was 21.258 (95% CI, 14.336; 31.522). TPS assigns each sample into a diagnostic category linked with a specific ROHM, guiding clinical management.

## 1. Introduction

Urine cytology is a safe and cost-effective diagnostic test showing suboptimal sensitivity yet high specificity to diagnose urothelial cancer [1]. Reasons to perform it include the initial evaluation of unexplained hematuria, a history of occupational exposure, or the follow-up of patients with previous diagnosis of urothelial cancer [2]. Bladder cancer is the most prevalent urothelial malignancy, whereas upper urinary tract cancers are relatively rare [3,4]. The former most often presents as a non-muscle invasive disease, either of low or high grade. Most patients recur after therapy, while some progress to muscle-invasive bladder cancer [5,6].

The Paris System (TPS) for Reporting Urinary Cytology is a standardized, evidence-based system that is applicable for either voided or instrumented specimens, and also for specimens sampled from both the lower and upper urinary tract. It was developed to standardize reporting, facilitating the communication among pathologists and between pathologists and clinicians [7,8]. TPS focuses on the diagnosis that is the most clinically important, the high-grade urothelial carcinoma (HGUC). It comprises seven diagnostic categories: nondiagnostic, negative for high-grade urothelial carcinoma (NHGUC), atypical urothelial cells (AUC), suspicious for high-grade urothelial carcinoma (SHGUC), HGUC, low-grade urothelial neoplasm (LGUN), and other primary or secondary malignancies [7]. TPS also supports the use of ancillary techniques (e.g., UroVysion FISH) for indeterminate interpretations [7,9].

Since the implementation of TPS, no meta-analysis has been published to summarize the experience collected worldwide with this reporting system. The main outcomes of this study were to:

1. Calculate the pooled risk of high-grade malignancy (ROHM) of each of the categories of TPS.
2. Display the diagnostic accuracy of urine cytology reported with TPS, by:
  - Creating a pooled summary ROC (sROC) curve and subsequently estimating the pooled sensitivity and false-positive rate.
  - Calculating the pooled Diagnostic Odds Ratio (DOR).

## 2. Materials and Methods

### 2.1. Search Strategy

This meta-analysis was performed following the guidelines set by the Preferred Reporting Item for Systematic Review and Meta-Analysis (PRISMA) Statement [10]. We comprehensively searched the literature for articles reporting on TPS on four databases (PubMed, Embase, Scopus, Web of Science) until 30 August 2020, using the following search term: “Paris system” AND (urin* OR cytopathology OR cytology)”. The PubMed database search was updated to add any additional studies published until February 2021, using the same term. No filters were applied, such as text availability, article type, or publication date. Duplicates were removed using the Paperpile reference manager (https://paperpile.com/app) (accessed on 30 August 2020), while the remaining records were uploaded into the Rayan App (https://www.rayyan.ai/) (accessed on 30 August 2020) for title–abstract selection [11].

### 2.2. Study Selection

We constructed our review question using the mnemonic PIRD (Population; Index test, Reference test, Diagnosis of interest) [12], where the “diagnosis of interest” was HGUC or other malignancies. The following inclusion criteria were applied:

- Studies on humans;
- Original studies;
- Follow-up present;
- Results reported with TPS.

In addition, we excluded studies based on the following criteria:

- Review articles, conference abstracts, editorials, and case reports;
- Articles written in a language other than English;
- In vitro or animal studies;
- Inability to extract data;
- Potential data overlap with already included studies;
- All enrolled patients had cancer and/or all follow-up cases showed cancer (high selection bias).

Three authors (I.P.N, Z.K. and M.K.) independently selected all relevant articles, while any disagreements were resolved with a consensus. The study selection was first performed in a title–abstract fashion with Rayyan, followed by a full screening of all Rayyan-eligible articles.

### 2.3. Data Extraction

The following data were extracted on an Excel^®^ spreadsheet: first author, year, country, study design, study period, specimen type (voided, instrumental, or both), urine cytology location (upper, lower urinary tract, or both), cytopreparation type (conventional, liquid-based cytology (LBC), or both), time of TPS classification (at initial Dx, reclassification of cases reported with another system), clinical setting (initial Dx, surveillance, or both), reference standard (histology, follow-up cytology, or both), total number of included cases and cases with follow-up, and total number of included patients and patients with follow-up (Table 1). Data concerning the prevalence of high-grade malignancy were extracted for each of the categories of TPS; HGUC and other malignancies were grouped together under a single category, as many studies reported these results together. To calculate the ROHM, diagnoses of both HGUC and other malignancies with the reference standard were considered as positive outcomes. Lastly, true positive (TP), true negative (TN), false positive (FP), and false negative (FN) data were extracted from each study. For this analysis, “nondiagnostic” TPS interpretations were excluded. Cases with the interpretations “NHGUC”, “AUC”, and “LGUN” were considered as cytologically negative, whereas “SHGUC”, “HGUC”, and “other malignancies” were considered as cytologically positive. For the histologic follow-up, only high-grade malignancies (HGUC; other malignancies) were considered as positive outcomes. Thus, a case with a cytologic interpretation of “SHGUC” or “HGUC was regarded as TP when histology revealed HGUC or another malignancy (e.g., prostate carcinoma); if not (e.g., histology outcome was non-neoplastic or even LGUN), it was regarded as FP. Any disagreements of the authors were resolved by a consensus.

### 2.4. Study Quality Assessment

Study quality assessment was performed with the Quality Assessment of Diagnostic Accuracy Studies (QUADAS-2) tool, under the following domains: patient selection; index test; reference standard; and flow and timing [12,41]. Risk of bias was assessed as low, unclear, or high. Results are shown in Table S1.

### 2.5. Statistical Analysis

We performed a prevalence and a diagnostic accuracy meta-analysis. In the first, we calculated the pooled ROHMs of each TPS category, while in the second, we constructed the sROC curve and assessed the pooled DOR. For the prevalence meta-analysis, a random intercept logistic regression model was applied. Heterogeneity was measured with tau^2^, Q, and I^2^. I^2^ levels > 50% indicate at least moderate heterogeneity, while levels > 75% indicate high levels of heterogeneity [42]. In addition, a continuity correction of 0.5 was applied in studies with zero cell frequencies. The sROC curve was constructed using both a proportional hazards approach [43] and a bivariate model [44]; “sensitivity” was put on the vertical, while “false-positive rate” on the horizontal axis of the curve. The Area Under the Curve (AUC) was then calculated to evaluate the discriminatory power of urine cytology reported with TPS. AUC values normally range from 0.5 (no discrimination) to 1 (perfect test) [45]. The log DOD of the index test was also calculated using the extracted TP, TN, FP, and FN data from each eligible study, using a random effects model. To investigate potential causes of heterogeneity, subgroup analyses were performed for the variables “specimen type”, “urine cytology location”, and “cytopreparation type”. Furthermore, sensitivity analyses were performed for the variables “study design”, “time of TPS classification”, and “follow-up type”. The analysis was performed with R, version 4.0.3 (R Foundation for Statistical Computing, Vienna, Austria).

## 3. Results

### 3.1. Literature Search

**Figure 1:**
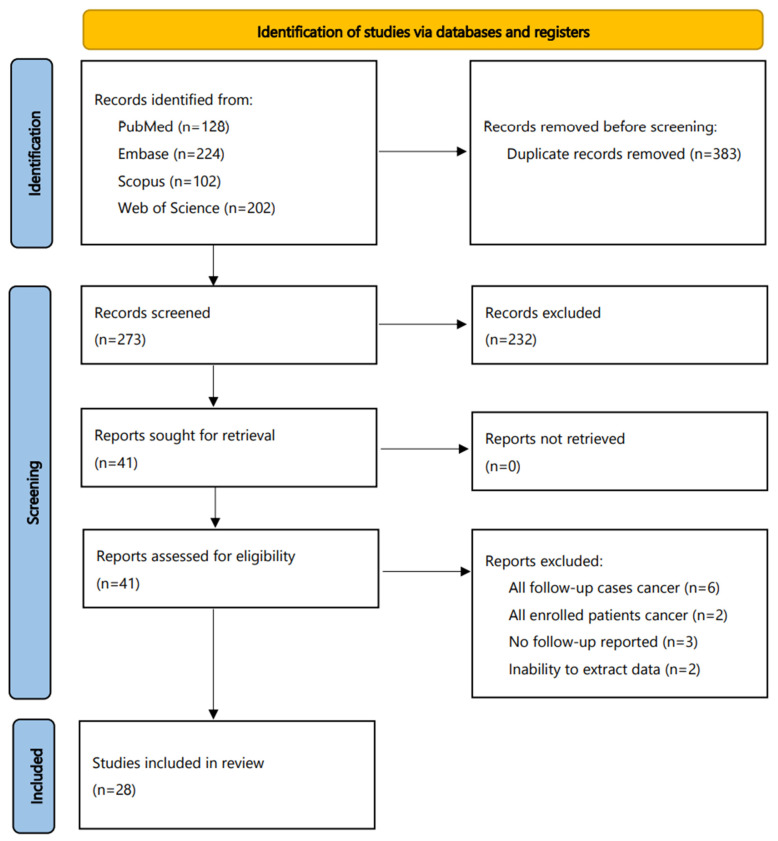
Flowchart of this meta-analysis.

The flowchart of this meta-analysis is shown in Figure 1. The initial search identified 644 studies (PubMed, 116; Embase, 224; Scopus, 102; Web of Science, 202), of which 383 were duplicates. The additional PubMed search added 12 more studies, resulting in a total 273 articles for screening in a title–abstract fashion. Of them, 41 were considered as eligible for full-text evaluation. After excluding 13 more articles at this step, 28 articles were included in this review. Whereas all 28 studies were included in the ROHM analyses, only 23 of them—with adequate data to create 2 × 2 contingency tables—were used for the diagnostic accuracy analyses.

### 3.2. Characteristics of the Included Studies

The main characteristics of the included studies are shown in Table 1. All studies were published between 2016 and 2021, and were performed worldwide, most commonly in the USA (*n* = 11). All but one had a retrospective design. The study period ranged from 1 year to 10 years and 5 months. Most studies examined both voided and instrumented samples (*n* = 15), from both the lower and upper urinary tract (*n* = 11), while they were processed with LBC (*n* = 15) rather than conventional cytology (*n* = 10). Less studies used TPS at the time of initial diagnosis (*n* = 12), whereas most reclassified their initial reported results to TPS for their particular study (*n* = 16). Follow-up was mainly provided by histology (*n* = 25), while three studies used both histology and follow-up cytology (*n* = 3). In the risk of bias evaluation (Table S1), no study was considered of low risk in all four QUADAS-2 domains. For instance, in the “patient selection” domain, some of the studies considered as having a high risk of bias used the number of cases with follow-up, rather than patients, for their analysis (some patients had more than one case). In the “reference standard” domain, the studies were considered to be of unclear bias, as histology was most likely performed with the knowledge of the index test (urine cytology) results. In addition, in the “Flow and Timing” domain, the three studies that used a different reference standard among their cases [13,14,40] were considered as having a high bias risk.

### 3.3. ROHM of the Categories of TPS

Table 2 shows the pooled ROHM associated with each of TPS categories. This was 17.70% for the nondiagnostic category (95% CI, 0.0650; 0.3997), 13.04% for the NHGUC (95% CI, 0.0932; 0.1796), 38.65% for the AUC (95% CI, 0.3042; 0.4759), 12.45% for the LGUN (95% CI, 0.0431; 0.3101), 76.89 for the SHGUC (95% CI, 0.7063; 0.8216), and 91.79% for the HGUC and other malignancies (95% CI, 0.8722; 0.9482). Heterogeneity was moderate to high for all TPS categories. Notably, when the risks were compared between studies that used LBC versus the ones used conventional cytology, no significant differences were found except for the category “nondiagnostic”; this had a ROHM of 6.41% (95% CI, 0.0181; 0.2035) in LBC and of 50.00% (95% CI, 0.3228; 0.6772) in conventional cytology (Tables S2 and S3).

### 3.4. Diagnostic Accuracy of Urine Cytology, Using TPS

**Figure 2:**
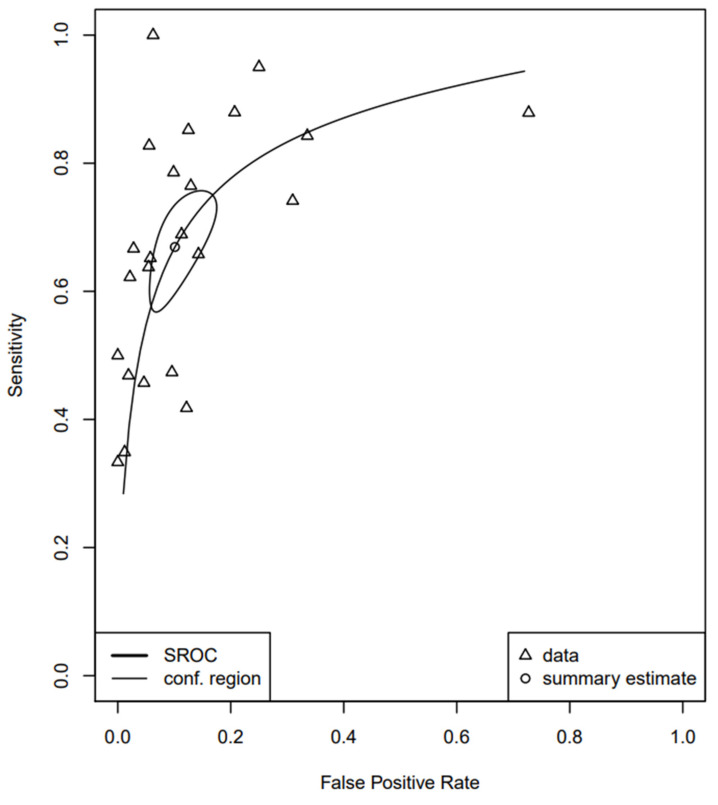
SROC curve of the included studies.

**Figure 3:**
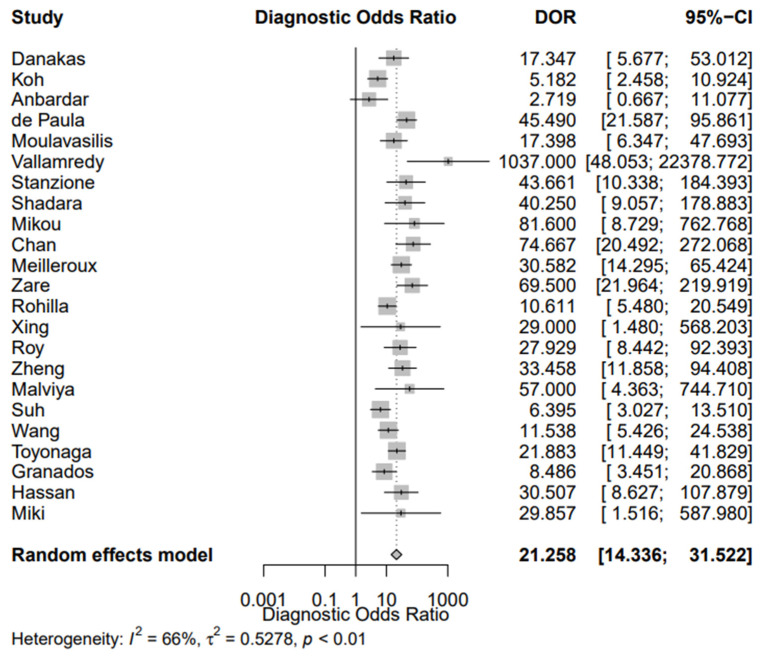
Diagnostic Odds Ratio (DOR) of the eligible studies.

**Figure 4:**
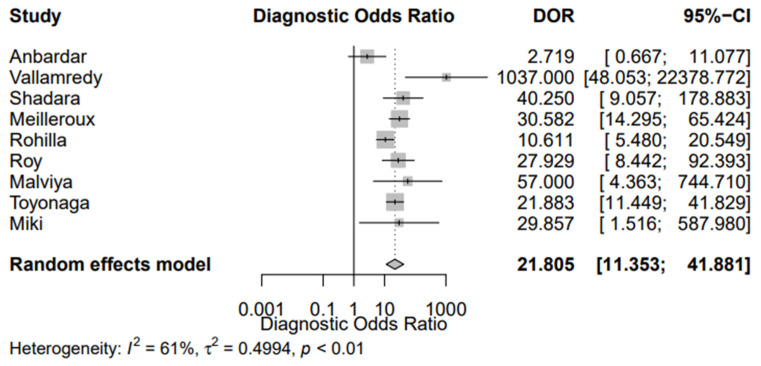
Diagnostic Odds Ratio (DOR) of the eligible studies using conventional cytology.

**Figure 5:**
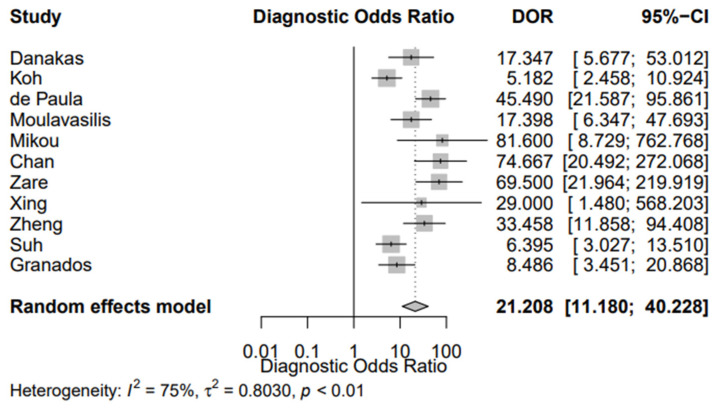
Diagnostic Odds Ratio (DOR) of the eligible studies using liquid-based cytology.

Figure 2 shows the sROC of the included studies, constructed with both the proportional hazards model approach and the bivariate model, respectively. The AUC was 0.849, while the pooled sensitivity was 0.669 (95% CI, 0.589; 0.741) and the false-positive rate was 0.101 (95% CI, 0.063; 0.158). In addition, the DOR of the included studies was 21.258 (95% CI, 14.336; 31.522) (Figure 3). Of interest, the DOR of conventional cytology (21.805 (95% CI, 11.353; 41.881)) was almost identical with that of LBC (21.208 (95% CI, 11.180; 40.228)) (Figure 4 and Figure 5).

## 4. Discussion

TPS is a standardized reporting system that facilitates communication among physicians and guides urology patients’ clinical management [1,7]. From its implementation, it has been shown to enhance correlation with histology, especially when the low urinary tract is sampled, while decreasing the indeterminate diagnoses [46,47]. Indeed, a few studies have demonstrated that TPS has reduced the rate of atypical interpretations reported in their departments [48,49,50,51]. This finding has a great clinical significance, as before the implementation of TPS, many urologists were regarding atypical cases as negative [6]. However, to enhance its sensitivity, some points for future TPS improvement have been pointed out, including the description of the hypochromatic HGUC [52], low-n/c-ratio HGUC [53], and plasmacytoid and micropapillary HGUC variants [54], besides the redefining the diagnostic criteria for the upper urinary tract, as the current ones miss a few positive cases [53,55].

This study first aimed to calculate the pooled ROHM of the categories of TPS. We combined data from all eligible studies published until February 2021. The ROHM ranged from 13.04% (95% CI, 0.0932; 0.1796) for the NHGUC to 91.79% (95% CI, 0.8722; 0.9482) for the HGUC and other malignancies. Notably, the ROHM for the AUC category was calculated at 38.65% (95% CI, 0.3042; 0.4759), prompting a close follow-up and potential ancillary testing with FISH or other modalities, such as UroSEEK, to better stratify such cases [1,9,56,57]. One reason why the ROHM of the SHGUC and HGUC categories was not closer to 100% could be the tendency of cytopathologists to overestimate the N/C ratio, as has been reported in the literature [58,59].

Our study also aimed to assess the diagnostic accuracy of urine cytology using TPS. We used the ROC method as our primary analysis, from which we calculated the AUC, in addition to the pooled sensitivity and false-positive rate. The AUC was 0.849, while the pooled sensitivity was 0.669 (95% CI, 0.589; 0.741). Two meta-analyses concerning the diagnostic performance of urine cytology have been published, combining the data published before the publication of TPS [7]. Xie et al. reported the pooled sensitivity of cytology detecting bladder cancer was 0.37 (95% CI, 0.35; 0.39), while the AUC was 0.80 [60]. Luo et al. specified their analysis on LBC and noted the pooled sensitivity was 0.58 (95% CI, 0.51; 0.65) and AUC 0.83 [61]. Both these meta-analyses pooled data from studies published before the implementation of TPS; in contrast, we included only TPS-based articles. We also found that the DOR of conventional cytology was 21.805 (95% CI, 11.353; 41.881), being almost identical with that of LBC (21.208 (95% CI, 11.180; 40.228)). Morphology of HGUC has been reported to be similar between conventional cytology and LBC [62]. Furthermore, they have not shown a significant difference concerning their sensitivity and specificity for diagnosing SHGUC or HGUC [63].

This study has some important limitations. Most studies were of small size, retrospective in nature, and with variability in their follow-up periods. A few of the eligible studies showed high risk of bias, especially in the “patient selection” domain of the QUADAS-2 tool. In addition, there was verification bias as the reference test was histology, which most likely enhanced the sensitivity and the ROHM in the nondiagnostic, NHGUC, and AUC categories [64,65]. As with most meta-analyses of diagnostic accuracy, our study also exhibited significant heterogeneity [12]. We applied subgroup and sensitivity analysis to assess the effect of a few variables, yet were unable to define its cause.

Academic cytopathologists have studied and debated the use of TPS, which is also a common topic at society meetings. However, general pathologists signing out cytopathology as well as clinicians may question the value of this new classification, since it seemingly has few differences compared to the conventional four-tiered system (“negative”; “atypical”, “suspicious”, and “positive”) most often used before the implementation of TPS. This metanalysis—the first one evaluating the diagnostic performance of urine cytology with TPS and assigning a pooled ROHM for each one of its reporting categories, guiding clinical management—could help them understand the general benefit of this evidence and consensus-based classification system. For example, many urologists before the implementation of TPS tended to regard “atypical” urine cytology as negative, as this interpretation was being used very often by pathologists [6]. Nevertheless, TPS focuses on what is more important, which is the detection of HGUC [1,7]. Thus, it has established strict criteria for each one of its categories, including AUC, aiming to identify HGUC rather than LGUN, resulting in a frequency reduction in the “atypical” interpretations compared to the pre-TPS era [48,49,50,51]. Of interest, the pooled ROHM of the AUC reporting category in our meta-analysis was found to be 38.65% (95% CI, 0.3042; 0.4759), which should warrant close clinical follow-up and/or the use of ancillary testing [1,7], rather than being regarded as negative.

## 5. Conclusions

We performed a meta-analysis to calculate a pooled ROHM for each TPS category and the diagnostic accuracy of urine cytology while applying this system. We hope our findings will be useful to pathologists and guide clinicians to select the best management plan for their patients.

## Figures and Tables

**Table 1:** Main characteristics of the studies included in the meta-analysis.

First Author/Year/Reference	Study Period	Country	Specimen Type	Lower vs. Upper Tract	Cytopreparation Type	Initial Dx or Reclassification	Reference Standard	Total Cases	Cases with Follow-Up
Abro, 2021 [13]	3 years	USA	Voided and Instrumented	Lower and Upper	LBC	Initial	Histology and follow-up cytology	230	116
McIntire, 2021 [14]	2 years	USA	Voided and Instrumented	Lower and Upper	LBC	Initial	Histology and follow-up cytology	2960	2960
Danakas, 2021 [15]	2 years	USA	Voided and Instrumented	NR	LBC	Initial	Histology	170	170
Nguyen, 2020 [16]	3 years, 7 months	USA	Voided and Instrumented	Lower and Upper	LBC	Initial	Histology	189	189
Koh, 2020 [17]	2 years	Korea	Voided	Lower	LBC	Reclassification	Histology	299	299
Anbardar, 2020 [18]	2 years, 6 months	Iran	Voided	Lower	Conventional	Reclassification	Histology	1842	55
Kuan, 2020 [19]	10 years, 5 months	USA	Voided and Instrumented	NR	Conventional	Initial	Histology	378	378
de Paula, 2020 [20]	2 years	Brazil	Voided and Instrumented	NR	LBC	Initial	Histology	1660	611
Moulavasilis, 2020 [21]	1 year	Greece	Voided and Instrumented	Lower	LBC	Initial	Histology	110	110
Vallamredy, 2019 [22]	5 years	India	NR	NR	Conventional	Reclassification	Histology	74	74
Stanzione, 2019 [23]	2 years, 7 months	USA	Voided and Instrumented	NR	NR	Initial	Histology	3202	294
Rai, 2019 [24]	1 year	India	NR	NR	Conventional	Initial	Histology	90	60
Mikou, 2018 [25]	1 year	Greece	Voided	Lower	LBC	Reclassification	Histology	720	47
Chan, 2018 [26]	6 years	USA	Voided and Instrumented	Lower and Upper	LBC	Reclassification	Histology	188	188
Meilleroux, 2018 [27]	2 years	France	Voided	Lower and Upper	Conventional	Initial	Histology	1814	299
Zare, 2018 [28]	2 years	USA	Voided and Instrumented	Lower	LBC	Reclassification	Histology	194	194
Rohilla, 2018 [29]	2 years	India	Voided	Lower and Upper	Conventional	Reclassification	Histology	4188	244
Xing, 2018 [30]	NR	USA	Instrumented	Upper	LBC	Reclassification	Histology	30	30
Roy, 2017 [31]	10 months	India	Voided	Lower and Upper	Conventional	Reclassification	Histology	255	97
Zheng, 2017 [32]	3 years, 4 months	USA	Instrumented	Upper	LBC	Reclassification	Histology	324	125
Malviya, 2017 [33]	1 year	India	Voided and Instrumented	Lower and Upper	Conventional	Reclassification	Histology	176	34
Suh, 2017 [34]	3 years	Korea	Instrumented	Lower and Upper	LBC	Reclassification	Histology	142	142
Wang, 2017 [35]	1 year	Canada	Voided and Instrumented	NR	LBC and Conventional	Initial	Histology	2392	167
Toyonaga, 2017 [36]	5 years, 8 months	Japan	Voided	Lower	Conventional	Reclassification	Histology	287	287
Granados, 2016 [37]	3 years	Spain	Voided	NR	LBC	Reclassification	Histology	149	149
Hassan, 2016 [38]	3 years	Canada	Voided and Instrumented	Lower	LBC and Conventional	Reclassification	Histology	124	124
Miki, 2016 [39]	6 years	UK	Voided and Instrumented	Lower and Upper	Conventional	Reclassification	Histology	91	45
Joudi, 2016 [40]	11 years	USA	Voided and Instrumented	Lower and Upper	LBC	Initial	Histology and follow-up cytology	662	662

Abbreviations: LBC, liquid-based cytology; NR, not reported.

**Table 2:** Pooled risk of high-grade malignancy (ROHM) associated with each of the Paris System categories.

Paris System Categories	No of Studies Pooled	ROHM (%)	95% CI	Tau^2^	Q	I^2^ (%)
Nondiagnostic	11	17.70	(0.0650; 0.3997)	1.8070	29.22	72.6
NHGUC	24	13.04	(0.0932; 0.1796)	0.6056	355.67	87.3
AUC	23	38.65	(0.3042; 0.4759)	0.5272	84.57	76.4
LGUN	10	12.45	(0.0431; 0.3101)	1.1790	4.89	55.4
SHGUC	26	76.89	(0.7063; 0.8216)	0.3291	53.12	66.1%
HGUC and other malignancies	25	91.79	(0.8722; 0.9482)	0.8732	92.36	82.6

Abbreviations: CI, confidence interval; NHGUC, negative for high-grade urothelial carcinoma; AUC, atypical urothelial cells; LGUC, low-grade urothelial neoplasm; SHGUC, suspicious for high-grade urothelial carcinoma; HGUC, high-grade urothelial carcinoma.

## Data Availability

Data are contained within the article or supplementary material.

## Supplementary Materials

The following supporting information can be downloaded at: https://www.mdpi.com/article/10.3390/jpm12020170/s1. Table S1: Risk of Bias of the studies included in the meta-analysis, according to the Quality Assessment of Diagnostic Accuracy Studies 2 (QUADAS-2). Table S2. Pooled risk of high-grade malignancy (ROHM) associated with each of the Paris System categories. Subgroup analysis of the studies using solely liquid-based cytology (LBC). Table S3. Pooled risk of high-grade malignancy (ROHM) associated with each of the Paris System categories. Subgroup analysis of the studies using solely conventional cytology.

## References

[1] Xing J., Reynolds J.P. (2018). Diagnostic Advances in Urine Cytology. Surg. Pathol. Clin..

[2] Van den Bussche C.J. (2016). A Review of the Paris System for Reporting Urinary Cytology. Cytopathology.

[3] Sung H., Ferlay J., Siegel R.L., Laversanne M., Soerjomataram I., Jemal A., Bray F. (2021). Global Cancer Statistics 2020: GLOBOCAN Estimates of Incidence and Mortality Worldwide for 36 Cancers in 185 Countries. CA Cancer J. Clin..

[4] Soualhi A., Rammant E., George G., Russell B., Enting D., Nair R., Van Hemelrijck M., Bosco C. (2021). The Incidence and Prevalence of Upper Tract Urothelial Carcinoma: A Systematic Review. BMC Urol..

[5] Chamie K., Litwin M.S., Bassett J.C., Daskivich T.J., Lai J., Hanley J.M., Konety B.R., Saigal C.S. (2013). Urologic Diseases in America Project Recurrence of High-Risk Bladder Cancer: A Population-Based Analysis. Cancer.

[6] Gupta M., VandenBussche C.J., Bivalacqua T.J. (2018). Urinary Cytology and the Paris System for Reporting Urinary Cytology: Implications for Urological Management. Cytopathology.

[7] Barkan G.A., Wojcik E.M., Nayar R., Savic-Prince S., Quek M.L., Kurtycz D.F.I., Rosenthal D.L. (2016). The Paris System for Reporting Urinary Cytology: The Quest to Develop a Standardized Terminology. J. Am. Soc. Cytopathol..

[8] Cowan M.L., VandenBussche C.J. (2018). The Paris System for Reporting Urinary Cytology: Early Review of the Literature Reveals Successes and Rare Shortcomings. J. Am. Soc. Cytopathol..

[9] Vlajnic T., Gut A., Savic S., Bubendorf L. (2019). The Paris System for Reporting Urinary Cytology in Daily Practice with Emphasis on Ancillary Testing by Multiprobe FISH. J. Clin. Pathol..

[10] Page M.J., McKenzie J.E., Bossuyt P.M., Boutron I., Hoffmann T.C., Mulrow C.D., Shamseer L., Tetzlaff J.M., Akl E.A., Brennan S.E. (2021). The PRISMA 2020 Statement: An Updated Guideline for Reporting Systematic Reviews. Int. J. Surg..

[11] Ouzzani M., Hammady H., Fedorowicz Z., Elmagarmid A. (2016). Rayyan—A Web and Mobile App for Systematic Reviews. Syst. Rev..

[12] Campbell J.M., Klugar M., Ding S., Carmody D.P., Hakonsen S.J., Jadotte Y.T., White S., Munn Z. (2015). Diagnostic Test Accuracy: Methods for Systematic Review and Meta-Analysis. Int. J. Evid. Based Healthc..

[13] Abro S., Nomani L., Wojcik E.M., Pambuccian S.E., Chatt G., Barkan G.A. (2021). Outcome Analysis and Negative Predictive Value of the “Unsatisfactory/nondiagnostic” Category of The Paris System for Reporting Urinary Cytology. J. Am. Soc. Cytopathol..

[14] McIntire P.J., Kilic I., Pambuccian S.E., Wojcik E.M., Barkan G.A. (2021). The Paris System for Reporting Urinary Cytology Reduces Atypia Rates and Does Not Alter the Negative Predictive Value of Urine Cytology. J. Am. Soc. Cytopathol..

[15] Danakas A., Sweeney M., Cheris S., Agrawal T. (2021). Urinary Tract Cytology: A Cytologic-Histopathologic Correlation with the Paris System, an Institutional Study. J. Am. Soc. Cytopathol..

[16] Nguyen L., Nilforoushan N., Krane J.F., Bose S., Bakkar R. (2020). Should “Suspicious for High-Grade Urothelial Carcinoma” and “Positive for High-Grade Urothelial Carcinoma” Remain Separate Categories?. Cancer Cytopathol..

[17] Koh H.H., Lee M.J., Park N.J., Kim H.-S., Oh Y.L. (2020). Impact of Implementing the Paris System for Reporting Urinary Cytology: A Single-Institutional Experience with Emphasis on Diagnostic Yield of High-Grade Urothelial Carcinoma and Low-Grade Urothelial Neoplasm. Anticancer Res..

[18] Anbardar M.H., Monjazeb R. (2020). Reclassification of Urinary Cytology Regarding the Paris System for Reporting Urinary Cytology with Cytohistological Correlation Demonstrates High Sensitivity for High-Grade Urothelial Carcinoma. Diagn. Cytopathol..

[19] Kuan K.C., Segura S.E., Ahlstedt J., Khader S.N., Hakima L. (2020). The Predictive Value of Positive and Suspicious Urine Cytology: Are They Different?. Diagn. Cytopathol..

[20] de Paula R., Oliveira A., Nunes W., Bovolim G., Domingos T., De Brot L., Bezerra S., Cunha I., Morini M., Saieg M. (2020). Two-Year Study on the Application of the Paris System for Urinary Cytology in a Cancer Centre. Cytopathology.

[21] Moulavasilis N., Lazaris A., Katafigiotis I., Stravodimos K., Constantinides C., Mikou P. (2020). Risk of Malignancy Assessment for the Paris System for Reporting Urinary Cytology. Diagn. Cytopathol..

[22] Begam K.V., Vallamreddy S.K.R., Pratima J. (2019). Implementation of the Paris System versus Institutional Diagnosis in the Performance of Urinary Cytology: A 5 Years Correlative Study of 74 Cases. IP Arch. Cytol. Histopathol. Res..

[23] Stanzione N., Ahmed T., Fung P.C., Cai D., Lu D.Y., Sumida L.C., Moatamed N.A. (2019). The Continual Impact of the Paris System on Urine Cytology, a 3-Year Experience. Cytopathology.

[24] Rai S., Lali B.S., Venkataramana C.G., Philipose C.S., Rao R., Prabhu G.L. (2019). A Quest for Accuracy: Evaluation of The Paris System in Diagnosis of Urothelial Carcinomas. J. Cytol..

[25] Mikou P., Lenos M., Papaioannou D., Vrettou K., Trigka E.-A., Sousouris S., Constantinides C. (2018). Evaluation of the Paris System in Atypical Urinary Cytology. Cytopathology.

[26] Chan E., Balassanian R., Tabatabai Z.L., Lou H., Vohra P. (2018). Improved Diagnostic Precision of Urine Cytology by Implementation of The Paris System and the Use of Cell Blocks. Cancer Cytopathol..

[27] Meilleroux J., Daniel G., Aziza J., d’Aure D.M., Quintyn-Ranty M.-L., Basset C.M.L., Evrard S.M., Courtade-Saidi M.M. (2018). One Year of Experience Using the Paris System for Reporting Urinary Cytology. Cancer Cytopathol..

[28] Zare S., Mirsadraei L., Reisian N., Liao X., Roma A., Shabaik A., Hasteh F. (2018). A Single Institutional Experience with the Paris System for Reporting Urinary Cytology: Correlation of Cytology and Histology in 194 Cases. Am. J. Clin. Pathol..

[29] Rohilla M., Singh P., Rajwanshi A., Gupta N., Srinivasan R., Dey P., Kakkar N. (2018). Cytohistological Correlation of Urine Cytology in a Tertiary Centre with Application of the Paris System. Cytopathology.

[30] Xing J., Monaco S.E., Pantanowitz L. (2018). Utility of the Paris System for Reporting Urinary Cytology in Upper Urinary Tract Specimens. J. Am. Soc. Cytopathol..

[31] Roy M., Kaushal S., Jain D., Seth A., Iyer V.K., Mathur S.R. (2017). An Institutional Experience with The Paris System: A Paradigm Shift from Ambiguous Terminology to More Objective Criteria for Reporting Urine Cytology. Cytopathology.

[32] Zheng X., Si Q., Du D., Harshan M., Zhang Z., Haines K., Shi W., Chhieng D.C. (2018). The Paris System for Urine Cytology in Upper Tract Urothelial Specimens: A Comparative Analysis with Biopsy and Surgical Resection. Cytopathology.

[33] Malviya K., Fernandes G., Naik L., Kothari K., Agnihotri M. (2017). Utility of the Paris System in Reporting Urine Cytology. Acta Cytol..

[34] Suh J., Go H., Sung C., Baek S., Hwang H., Jeong S., Cho Y. (2017). Modification of The Paris System for Urinary Tract Washing Specimens Using Diagnostic Cytological Features. Cytopathology.

[35] Wang Y., Auger M., Kanber Y., Caglar D., Brimo F. (2017). Implementing the Paris System for Reporting Urinary Cytology Results in a Decrease in the Rate of the “atypical” Category and an Increase in Its Prediction of Subsequent High-Grade Urothelial Carcinoma. Cancer Cytopathol..

[36] Toyonaga Y., Yamazaki K., Koyama Y., Yamada M., Ishida Y. (2017). A Modified Direct-Smear Processing Technique Employing Two-Step Centrifugation/Fixation Is Useful for Detecting High-Grade Urothelial Carcinoma. Acta Cytol..

[37] Granados R., Duarte J.A., Corrales T., Camarmo E., Bajo P. (2016). Applying the Paris System for Reporting Urine Cytology Increases the Rate of Atypical Urothelial Cells in Benign Cases: A Need for Patient Management Recommendations. Acta Cytol..

[38] Hassan M., Solanki S., Kassouf W., Kanber Y., Caglar D., Auger M., Brimo F. (2016). Impact of Implementing the Paris System for Reporting Urine Cytology in the Performance of Urine Cytology: A Correlative Study of 124 Cases. Am. J. Clin. Pathol..

[39] Miki Y., Neat M., Chandra A. (2016). Application of The Paris System to Atypical Urine Cytology Samples: Correlation with Histology and UroVysion (R) FISH. Cytopathology.

[40] Joudi A.M., Pambuccian S.E., Wojcik E.M., Barkan G.A. (2016). The Positive Predictive Value of “Suspicious for High-Grade Urothelial Carcinoma” in Urinary Tract Cytology Specimens: A Single-Institution Study of 665 Cases. Cancer Cytopathol..

[41] Whiting P.F., Rutjes A.W.S., Westwood M.E., Mallett S., Deeks J.J., Reitsma J.B., Leeflang M.M.G., Sterne J.A.C., Bossuyt P.M.M. (2011). QUADAS-2 Group QUADAS-2: A Revised Tool for the Quality Assessment of Diagnostic Accuracy Studies. Ann. Intern. Med..

[42] Higgins J.P.T., Thompson S.G., Deeks J.J., Altman D.G. (2003). Measuring Inconsistency in Meta-Analyses. BMJ.

[43] Holling H., Böhning W., Böhning D. (2012). Meta-Analysis of Diagnostic Studies Based upon SROC-Curves: A Mixed Model Approach Using the Lehmann Family. Stat. Model..

[44] Reitsma J.B., Glas A.S., Rutjes A.W.S., Scholten R.J.P.M., Bossuyt P.M., Zwinderman A.H. (2005). Bivariate Analysis of Sensitivity and Specificity Produces Informative Summary Measures in Diagnostic Reviews. J. Clin. Epidemiol..

[45] Jones C.M., Athanasiou T. (2005). Summary Receiver Operating Characteristic Curve Analysis Techniques in the Evaluation of Diagnostic Tests. Ann. Thorac. Surg..

[46] McIntire P.J., Snow J.T., Robinson B.D., Rao R.A., Goyal A., Heymann J.J., Siddiqui M.T. (2018). Improved Correlation of Urinary Cytology Specimens Using the Paris System in Biopsy-Proven Upper Tract Urothelial Carcinomas. Cancer Cytopathol..

[47] Bertsch E.C., Siddiqui M.T., Ellis C.L. (2018). The Paris System for Reporting Urinary Cytology Improves Correlation with Surgical Pathology Biopsy Diagnoses of the Lower Urinary Tract. Diagn. Cytopathol..

[48] Torous V.F., Brancely D., VanderLaan P.A. (2017). Implementation of the Paris System for Reporting Urinary Cytology Results in Lower Atypical Diagnostic Rates. J. Am. Soc. Cytopathol..

[49] Hang J.-F., Charu V., Zhang M.L., VandenBussche C.J. (2017). Digital Image Analysis Supports a Nuclear-to-Cytoplasmic Ratio Cutoff Value of 0.5 for Atypical Urothelial Cells. Cancer Cytopathol..

[50] Rohra P., Ocampo Gonzalez F.A., Yan L., Mir F., Furlan K., Basu S., Barua A., Cheng L., Park J.-W. (2021). Effect of the Paris System for Reporting Urinary Cytology with Histologic Follow-Up. Diagn. Cytopathol..

[51] Tian W., Shore K.T., Shah R.B. (2021). Significant Reduction of Indeterminate (atypical) Diagnosis after Implementation of The Paris System for Reporting Urinary Cytology: A Single-Institution Study of More than 27,000 Cases. Cancer Cytopathol..

[52] Pierconti F., Martini M., Straccia P., Fiorentino V., Musarra T., Larocca L.M., Lopez-Beltran A. (2018). Hypochromatic Large Urothelial Cells in Urine Cytology Are Indicative of High Grade Urothelial Carcinoma. APMIS.

[53] Kurtycz D.F.I., Sundling K.E., Barkan G.A. (2020). The Paris System of Reporting Urinary Cytology: Strengths and Opportunities. Diagn. Cytopathol..

[54] Suo L., Vega I., Thrall M. (2021). Cyto-Histo Correlations of Plasmacytoid and Micropapillary Variants of High-Grade Urothelial Carcinoma: Do They Fit Well in The Paris System for Reporting Urinary Cytology?. J. Am. Soc. Cytopathol..

[55] Simon C.T., Skala S.L., Magers M.J., Weizer A., Kaffenberger S.D., Chinnaiyan A.M., Spratt D.E., Montgomery J., Mehra R., Lew M. (2018). The Utility of Upper Urinary Tract Urine Cytology before and after Application of the Paris System. Diagn. Cytopathol..

[56] Virk R.K., Abro S., de Ubago J.M.M., Pambuccian S.E., Quek M.L., Wojcik E.M., Mehrotra S., Chatt G.U., Barkan G.A. (2017). The Value of the UroVysion® FISH Assay in the Risk-Stratification of Patients with “atypical Urothelial Cells” in Urinary Cytology Specimens. Diagn. Cytopathol..

[57] Rodriguez Pena M.D.C., Springer S.U., Taheri D., Li L., Tregnago A.C., Eich M.-L., Eltoum I.-E.A., VandenBussche C.J., Papadopoulos N., Kinzler K.W. (2019). Performance of Novel Non-Invasive Urine Assay UroSEEK in Cohorts of Equivocal Urine Cytology. Virchows Arch..

[58] Zhang M.L., Guo A.X., VandenBussche C.J. (2016). Morphologists Overestimate the Nuclear-to-Cytoplasmic Ratio. Cancer Cytopathol..

[59] Wang Y.-H., Hang J.-F., Wen C.-H., Liao K.-C., Lee W.-Y., Lai C.-R. (2020). Diagnostic Agreement for High-Grade Urothelial Cell Carcinoma in Atypical Urine Cytology: A Nationwide Survey Reveals a Tendency for Overestimation in Specimens with an N/C Ratio Approaching 0.5. Cancers.

[60] Xie Q., Huang Z., Zhu Z., Zheng X., Liu J., Zhang M., Zhang Y. (2016). Diagnostic Value of Urine Cytology in Bladder Cancer. A Meta-Analysis. Anal. Quant. Cytopathol. Histpathol..

[61] Luo Y., She D.-L., Xiong H., Yang L., Fu S.-J. (2015). Diagnostic Value of Liquid-Based Cytology in Urothelial Carcinoma Diagnosis: A Systematic Review and Meta-Analysis. PLoS ONE.

[62] Richardson C.J., Pambuccian S.E., Barkan G.A. (2020). Split-Sample Comparison of Urothelial Cells in ThinPrep and Cytospin Preparations in Urinary Cytology: Do We Need to Adjust the Paris System for Reporting Urinary Cytology Criteria?. Cancer Cytopathol..

[63] Straccia P., Bizzarro T., Fadda G., Pierconti F. (2016). Comparison between Cytospin and Liquid-Based Cytology in Urine Specimens Classified according to the Paris System for Reporting Urinary Cytology. Cancer Cytopathol..

[64] Farahani S.J., Baloch Z. (2019). Retrospective Assessment of the Effectiveness of the Milan System for Reporting Salivary Gland Cytology: A Systematic Review and Meta-Analysis of Published Literature. Diagn. Cytopathol..

[65] Hoda R.S., Finer E.B., Arpin R.N., Rosenbaum M., Pitman M.B. (2019). Risk of Malignancy in the Categories of the Papanicolaou Society of Cytopathology System for Reporting Pancreaticobiliary Cytology. J. Am. Soc. Cytopathol..

