# Supplementary material for: The Paris System for Reporting Urinary Cytology: A Meta-Analysis
Source: J Pers Med. 2022 Jan 27;12(2):170. doi: 10.3390/jpm12020170 (PMC8874476; doi:10.3390/jpm12020170)
Supplement: Supplementary file 1 [file jpm-12-00170-s001.zip › jpm-1547308-SI.pdf]

Table S1. Risk of Bias of the studies included in the meta-analysis, according to the Quality Assessment of Diagnostic Accuracy Studies 2 (QUADAS-2).

First Author/ Reference	Patient Selection	Index Test	Reference Standard	Flow and Timing
Abro [1]	H	L	U	H
McIntire [2]	H	L	U	H
Danakas [3]	L	L	U	L
Nguyen [4]	L	L	U	L
Koh [5]	L	L	U	L
Anbardar [6]	H	L	U	L
Kuan [7]	H	L	U	L
de Paula [8]	L	L	U	L
Moulavasilis [9]	H	L	U	L
Vallamredy [10]	L	L	U	L
Stanzione [11]	L	L	U	L
Rai [12]	L	L	U	L
Mikou [13]	L	L	U	L
Chan [14]	L	L	U	L
Meilleroux [15]	U	L	U	L

Zare [16]	L	L	U	L
Rohilla [17]	L	L	U	L
Xing [18]	H	L	U	L
Roy [19]	L	L	U	L
Zheng [20]	H	L	U	L
Malviya [21]	L	L	U	L
Suh [22]	H	L	U	L
Wang [23]	L	L	U	L
Toyonaga [24]	L	U	U	L
Granados [25]	L	L	U	L
Hassan [26]	L	L	U	L
Miki [27]	H	L	U	L
Joudi [28]	H	L	U	H

Abbreviations: L, low; U, unclear; H, high

Table S2. Pooled risk of high-grade malignancy (ROHM) associated with each of the Paris System categories. Subgroup analysis of the studies using solely liquid-based cytology (LBC).

Paris System Categories	No of Studies Pooled	ROHM (%)	95% CI	tau^2	Q	I^2 (%)
**Nondiagnostic**	5	**6.41**	[0.0181; 0.2035]	0.7028	8.06	53.6
**NHGUC**	12	**12.82**	[0.0739; 0.2133]	0.9657	328.09	93.9

**AUC**	11	**40.48**	[0.2849; 0.5373]	0.5994	58.06	76.3
**LGUN**	4	**11.43**	[0.0436; 0.2677]	0	0.17	0.0
**SHGUC**	13	**77.49**	[0.6728; 0.8521]	0.5153	35.91	75.9
**HGUC and other malignancies**	12	**90.26**	[0.8513; 0.9375]	0.2471	15.92	50.1

Abbreviations: CI, confidence interval; NHGUC, negative for high-grade urothelial carcinoma; AUC, atypical urothelial cells; LGUC, low-grade urothelial neoplasm; SHGUC, suspicious for high-grade urothelial carcinoma; HGUC, high-grade urothelial carcinoma

Table S3. Pooled risk of high-grade malignancy (ROHM) associated with each of the Paris System categories. Subgroup analysis of the studies using solely conventional cytology.

**Paris System Categories**	**No of Studies Pooled**	**ROHM (%)**	**95% CI**	**tau^2**	**Q**	**I^2 (%)**
**Nondiagnostic**	6	**50.00**	[0.3228; 0.6772]	0	0.35	0.0
**NHGUC**	9	**13.41**	[0.0955; 0.1851]	0.0517	6.52	16.6
**AUC**	9	**30.67**	[0.1790; 0.4729]	0.6567	23.01	70.6
**LGUN**	6	**14.77**	[0.0320; 0.4759]	1.6611	3.00	62.9
**SHGUC**	10	**74.06**	[0.6938; 0.7825]	0	11.28	0.0
**HGUC and other malignancies**	10	**91.82**	[0.8145; 0.9663]	1.3831	54.25	88.3

Abbreviations: CI, confidence interval; NHGUC, negative for high-grade urothelial carcinoma; AUC, atypical urothelial cells; LGUC, low-grade urothelial neoplasm; SHGUC, suspicious for high-grade urothelial carcinoma; HGUC, high-grade urothelial carcinoma

References

1. Abro, S.; Nomani, L.; Wojcik, E.M.; Pambuccian, S.E.; Chatt, G.; Barkan, G.A. Outcome Analysis and Negative Predictive Value of the "Unsatisfactory/nondiagnostic" Category of The Paris System for Reporting Urinary Cytology. *J. Am. Soc. Cytopathol.* **2021**, *10*, 64–70.
2. McIntire, P.J.; Kilic, I.; Pambuccian, S.E.; Wojcik, E.M.; Barkan, G.A. The Paris System for Reporting Urinary Cytology Reduces Atypia Rates and Does Not Alter the Negative Predictive Value of Urine Cytology. *J. Am. Soc. Cytopathol.* **2021**, *10*, 14–19.
3. Danakas, A.; Sweeney, M.; Cheris, S.; Agrawal, T. Urinary Tract Cytology: A Cytologic-Histopathologic Correlation with The Paris System, an Institutional Study. *J. Am. Soc. Cytopathol.* **2021**, *10*, 56–63.
4. Nguyen, L.; Nilforoushan, N.; Krane, J.F.; Bose, S.; Bakkar, R. Should "Suspicious for High-Grade Urothelial Carcinoma" and "Positive for High-Grade Urothelial Carcinoma" Remain Separate Categories? *Cancer Cytopathol.* **2020**. doi:10.1002/cncy.22357.
5. Koh, H.H.; Lee, M.J.; Park, N.J.; Kim, H.-S.; Oh, Y.L. Impact of Implementing the Paris System for Reporting Urinary Cytology: A Single-Institutional Experience With Emphasis on Diagnostic Yield of High-Grade Urothelial Carcinoma and Low-Grade Urothelial Neoplasm. *Anticancer. Res.* **2020**, *40*, 3477–3484.
6. Anbardar, M.H.; Monjazeb, R. Reclassification of Urinary Cytology Regarding The Paris System for Reporting Urinary Cytology with Cytohistological Correlation Demonstrates High Sensitivity for High-Grade Urothelial Carcinoma. *Diagn. Cytopathol.* **2020**, *48*, 446–452.
7. Kuan, K.C.; Segura, S.E.; Ahlstedt, J.; Khader, S.N.; Hakima, L. The Predictive Value of Positive and Suspicious Urine Cytology: Are They Different? *Diagn. Cytopathol.* **2020**. doi:10.1002/dc.24531.
8. de Paula, R.; Oliveira, A.; Nunes, W.; Bovolim, G.; Domingos, T.; De Brot, L.; Bezerra, S.; Cunha, I.; Morini, M.; Saieg, M. Two-Year Study on the Application of the Paris System for Urinary Cytology in a Cancer Centre. *Cytopathology* **2020**, *31*, 41–46.
9. Moulavasilis, N.; Lazaris, A.; Katafigiotis, I.; Stravodimos, K.; Constantinides, C.; Mikou, P. Risk of Malignancy Assessment for the Paris System for Reporting Urinary Cytology. *Diagn. Cytopathol.* **2020**. doi:10.1002/dc.24575.
10. Begam K, V.; Kota Reddy Vallamreddy, S.; Pratima, J. Implementation of the Paris System versus Institutional

Diagnosis in the Performance of Urinary Cytology: A 5 Years Correlative Study of 74 Cases. *IP Arch. Cytol. Histopathol. Res.* **2019**, *4*, 193–198.
11. Stanzione, N.; Ahmed, T.; Fung, P.C.; Cai, D.; Lu, D.Y.; Sumida, L.C.; Moatamed, N.A. The Continual Impact of the Paris System on Urine Cytology, a 3-Year Experience. *Cytopathology* **2019**, *31*, 35–40.
12. Rai, S.; Lali, B.S.; Venkataramana, C.G.; Philipose, C.S.; Rao, R.; Prabhu, G.L. A Quest for Accuracy: Evaluation of The Paris System in Diagnosis of Urothelial Carcinomas. *J. Cytol.* **2019**, *36*, 169–173.
13. Mikou, P.; Lenos, M.; Papaioannou, D.; Vrettou, K.; Trigka, E.-A.; Sousouris, S.; Constantinides, C. Evaluation of the Paris System in Atypical Urinary Cytology. *Cytopathology* **2018**, *29*, 545–549.
14. Chan, E.; Balassanian, R.; Tabatabai, Z.L.; Lou, H.; Vohra, P. Improved Diagnostic Precision of Urine Cytology by Implementation of The Paris System and the Use of Cell Blocks. *Cancer Cytopathol.* **2018**, *126*, 809–816.
15. Meilleroux, J.; Daniel, G.; Aziza, J.; d'Aure, D.M.; Quintyn-Ranty, M.-L.; Basset, C.M.L.; Evrard, S.M.; Courtade-Saidi, M.M. One Year of Experience Using the Paris System for Reporting Urinary Cytology. *Cancer Cytopathol.* **2018**, *126*, 430–436.
16. Zare, S.; Mirsadraei, L.; Reisian, N.; Liao, X.; Roma, A.; Shabaik, A.; Hasteh, F. A Single Institutional Experience with the Paris System for Reporting Urinary Cytology: Correlation of Cytology and Histology in 194 Cases. *Am. J. Clin. Pathol.* **2018**, *150*, 162–167.
17. Rohilla, M.; Singh, P.; Rajwanshi, A.; Gupta, N.; Srinivasan, R.; Dey, P.; Kakkar, N. Cytohistological Correlation of Urine Cytology in a Tertiary Centre with Application of the Paris System. *Cytopathology* **2018**, *29*, 436–443.
18. Xing, J.; Monaco, S.E.; Pantanowitz, L. Utility of The Paris System for Reporting Urinary Cytology in Upper Urinary Tract Specimens. *J. Am. Soc. Cytopathol.* **2018**, *7*, 311–317.
19. Roy, M.; Kaushal, S.; Jain, D.; Seth, A.; Iyer, V.K.; Mathur, S.R. An Institutional Experience with The Paris System: A Paradigm Shift from Ambiguous Terminology to More Objective Criteria for Reporting Urine Cytology. *Cytopathology* **2017**, *28*, 509–515.
20. Zheng, X.; Si, Q.; Du, D.; Harshan, M.; Zhang, Z.; Haines, K., 3rd; Shi, W.; Chhieng, D.C. The Paris System for Urine Cytology in Upper Tract Urothelial Specimens: A Comparative Analysis with Biopsy and Surgical Resection. *Cytopathology* **2018**, *29*, 184–188.
21. Malviya, K.; Fernandes, G.; Naik, L.; Kothari, K.; Agnihotri, M. Utility of the Paris System in Reporting Urine Cytology. *Acta Cytol.* **2017**, *61*, 145–152.

22. Suh, J.; Go, H.; Sung, C.; Baek, S.; Hwang, H.; Jeong, S.; Cho, Y. Modification of The Paris System for Urinary Tract Washing Specimens Using Diagnostic Cytological Features. *Cytopathology* **2017**, *28*, 516–523.
23. Wang, Y.; Auger, M.; Kanber, Y.; Caglar, D.; Brimo, F. Implementing The Paris System for Reporting Urinary Cytology Results in a Decrease in the Rate of the "atypical" Category and an Increase in Its Prediction of Subsequent High-Grade Urothelial Carcinoma. *Cancer Cytopathol.* **2017**, *126*, 207–214.
24. Toyonaga, Y.; Yamazaki, K.; Koyama, Y.; Yamada, M.; Ishida, Y. A Modified Direct-Smear Processing Technique Employing Two-Step Centrifugation/Fixation Is Useful for Detecting High-Grade Urothelial Carcinoma. *Acta Cytol.* **2017**, *61*, 447–454.
25. Granados, R.; Duarte, J.A.; Corrales, T.; Camarmo, E.; Bajo, P. Applying the Paris System for Reporting Urine Cytology Increases the Rate of Atypical Urothelial Cells in Benign Cases: A Need for Patient Management Recommendations. *Acta Cytol.* **2016**, *61*, 71–76.
26. Hassan, M.; Solanki, S.; Kassouf, W.; Kanber, Y.; Caglar, D.; Auger, M.; Brimo, F. Impact of Implementing the Paris System for Reporting Urine Cytology in the Performance of Urine Cytology: A Correlative Study of 124 Cases. *Am. J. Clin. Pathol.* **2016**, *146*, 384–390.
27. Miki, Y.; Neat, M.; Chandra, A. Application of The Paris System to Atypical Urine Cytology Samples: Correlation with Histology and UroVysion (R) FISH. *Cytopathology* **2016**, *28*, 88–95.
28. Joudi, A.M.; Pambuccian, S.E.; Wojcik, E.M.; Barkan, G.A. The Positive Predictive Value of "Suspicious for High-Grade Urothelial Carcinoma" in Urinary Tract Cytology Specimens: A Single-Institution Study of 665 Cases. *Cancer Cytopathol.* **2016**, *124*, 811–819.
